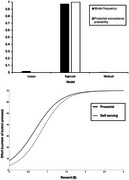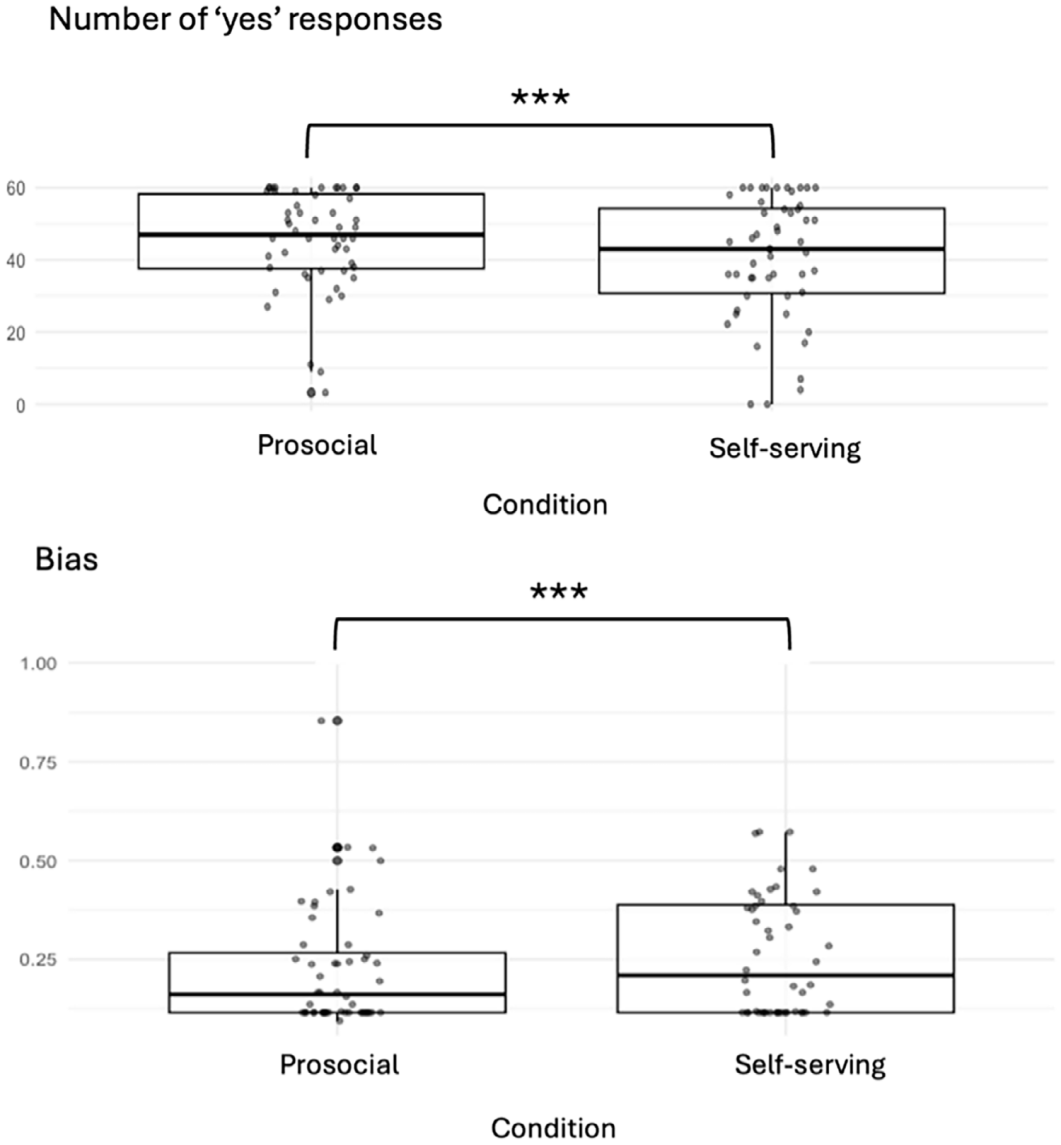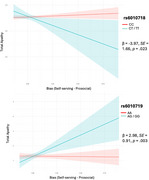# OPRL1 genotypes moderate the association between prosocial effort‐based decision making and apathy in at‐risk older adults

**DOI:** 10.1002/alz70857_106007

**Published:** 2025-12-26

**Authors:** Caitlin S. Walker, Garance Barnoin, Mitchell Bennett, Sylvia Villeneuve, Cynthia Picard, Judes Poirier, Maiya R. Geddes, the PREVENT‐AD Research Group

**Affiliations:** ^1^ Montreal Neurological Institute‐Hospital (The Neuro), McGill University, Montreal, QC, Canada; ^2^ McGill University, Montreal, QC, Canada; ^3^ Department of Neurology and Neurosurgery, McGill University, Montreal, QC, Canada; ^4^ Douglas Mental Health University Institute, Centre for Studies on the Prevention of Alzheimer's Disease (StoP‐AD), Montréal, QC, Canada; ^5^ Douglas Mental Health University Institute, Montreal, QC, Canada; ^6^ McConnell Brain Imaging Centre (BIC), MNI, Faculty of Medicine, McGill University, Montreal, QC, Canada; ^7^ StoP‐AD Centre, Douglas Mental Health Institute Research Centre, Montreal, QC, Canada; ^8^ Centre for Studies on Prevention of Alzheimer's disease (StoP‐AD Centre), Montreal, QC, Canada; ^9^ Department of Psychiatry, McGill University, Montréal, QC, Canada; ^10^ Centre for Studies on Prevention of Alzheimer's Disease (StoP‐AD Centre), Douglas Mental Health University Institute, Montréal, QC, Canada; ^11^ Centre for Studies in the Prevention of Alzheimer's Disease, Douglas Mental Health Institute, McGill University, Montreal, QC, Canada; ^12^ Department of Psychiatry, McGill University, Montreal, QC, Canada; ^13^ McGill University Research Centre for Studies in Aging, McGill University, Montreal, QC, Canada; ^14^ The Neuro, Faculty of Medicine, McGill University, Montreal, QC, Canada; ^15^ Centre for Studies on Prevention of Alzheimer's Disease (StoP‐AD Centre), Montreal, QC, Canada; ^16^ Massachusetts Institute of Technology, Cambridge, MA, USA; ^17^ Rotman Research Institute, University of Toronto, Toronto, ON, Canada

## Abstract

**Background:**

Apathy is a common neuropsychiatric symptom in early Alzheimer's disease (AD) characterized by reduced goal‐directed activity and motivation. Older adults tend to value prosocial over self‐serving behaviors, with this shift being preserved in early AD. It is unknown whether prosocial rewards are more effective at motivating effort and how prosocial orientations relate to apathy in at‐risk older adults. OPRL1 encodes the nociceptin/orphanin FQ receptor, which downregulates dopaminergic activity in the ventral tegmental area, a region critical for reward processing. Building on evidence that prosocial behaviors are inherently rewarding for older adults, we hypothesized that older adults would exert more effort for prosocial than self‐serving rewards and that OPRL1 genetic variations would modulate the relationship between prosocial orientations and apathy.

**Method:**

52 older adults (*M*_age_ = 68.48, 38 females) from the PREVENT‐AD cohort completed a novel computerized paradigm where they decided whether to exert effort (button presses) for prosocial (charity donations) and self‐serving (monetary) rewards. Reward‐effort discounting curves were modeled with linear, sigmoid, and Weibull functions, and random‐effects Bayesian model comparison identified the best fitting model. Effort willingness (“yes” responses) and amount of reward needed to initiate effort (bias) were compared between conditions, and difference scores quantified participants’ prosocial orientations. Regression models examined associations between difference and Apathy Motivation Index scores. Genotypes of OPRL1 rs6010718 and rs6010719 SNPs were included as moderators.

**Result:**

Sigmoid functions best fit reward‐effort discounting curves (Figure 1). Participants made more ‘yes’ responses and showed lower bias for prosocial compared to self‐serving rewards (Figure 2). For rs6010718, minor T allele carriers showed lower apathy associated with reduced bias for prosocial relative to self‐serving rewards, whereas CC individuals showed no effect (Figure 3). For rs6010719, minor G allele carriers showed higher apathy associated with reduced bias for prosocial relative to self‐serving rewards, while AA individuals showed no effect (Figure 3).

**Conclusion:**

Older adults at risk for AD demonstrate a higher willingness and require smaller rewards to exert effort for prosocial compared to self‐serving rewards. Genetic variations in OPRL1 SNPs modulate the relationship between prosocial orientations and apathy, suggesting individuals with specific genetic profiles show reduced apathetic behavior associated with prosocial effort in aging.